# Letter to the editor: Pneumatosis in bowel ischemia: time to change the optics to improve patient care

**DOI:** 10.1186/s13244-022-01165-z

**Published:** 2022-02-22

**Authors:** Paul Calame, Éric Delabrousse, Maxime Ronot

**Affiliations:** 1 Department of Radiology, University of Bourgogne Franche-Comté, CHRU Besançon, 25030 Besançon, France; 2 EA 4662 Nanomedicine Lab, Imagery and Therapeutics, University of Franche-Comté, Besançon, France; 3 Department of Radiology, University Hospitals Paris Nord Val-de-Seine, AP—HP, Beaujon, 92110 Clichy, France

**Keywords:** Tomography (X-ray computed), Mesenteric ischemia, Ischemia, Laparotomy, Pneumatosis cystoids intestinalis

## Key points

- CT features of bowel ischemia differ between occlusive and non-occlusive mesenteric ischemia.
- In NOMI, pneumatosis overdiagnoses necrosis. The absence of bowel wall enhancement and a thinned wall on CT better predict transmural necrosis.
- In occlusive AMI, necrosis is underdiagnosed if only pneumatosis is considered. More frequent features such as bowel dilation and should not be overlooked.
- Overall, pneumatosis is not a synonym of irreversible transmural necrosis and should not systematically lead to surgical exploration.

## Background

To the Editor in Chief,

We read with interest the article entitled ‘Many faces of acute bowel ischemia: overview of radiologic staging’ by Davarpanah A. et al. [1] recently published in *Insights into Imaging*.

The authors propose a stimulating review of all etiologies of acute bowel infarction (ABI) with compelling illustrations of correlations between pathologic and radiologic findings of ABI according to the three main stages of bowel ischemia (i.e., early, Intermediate, and late phase of ischemia). We congratulate the authors for their extensive overview, and we fully share their call for a better understanding of the pathophysiology of ABI to help improve patient management. However, we believe two points call for comments. First, the authors imply that radiologic and pathologic findings of the different ischemic stages are similar in NOMI and in occlusive forms of AMI, which is inaccurate. Second, they endorse the idea that a gas pattern is a specific finding of a late phase of ischemia, which is questionable and should be challenged.

## Main text

As stated by the authors, physiopathology, timeline, and contexts are very different between NOMI and occlusive forms of AMI. Occlusive AMI occurs in patients with a frequent history of cardiovascular comorbidities and presents as a time-dependent continuum between ischemia and necrosis that leads to organ failure and eventually death. NOMI classically occurs in critically ill patients. Bowel ischemia is often more diffuse and fluctuates over time, leading to different patterns of necrosis. Understandably, clinical, biological, but also radiological characteristics substantially differ between NOMI and occlusive AMI. While the distinction between early, intermediate, and late forms presented by the authors seems more suited to the pathophysiology of occlusive AMI, the development of necrosis remains the most important prognostic factor in all forms nonetheless. Therefore, the assessment of the probability of necrosis is of utmost importance. In this setting, bowel wall pneumatosis has been classically considered one of the most specific features on imaging.

Pneumatosis has a long medical story. First described as pneumatosis cystoid in 1730 and initially subject to confusion between diagnosis and symptom, its detection has been primarily favored by the widespread use of CT over the past decades. Due to its easily recognizable character and its striking semiology, the gas pattern has fascinated the medical community, especially radiologists, because of their first-line position in the diagnostic workup. It has been rapidly associated with late-stage AMI and necrosis, but also with many other benign conditions.

When dealing with pneumatosis in AMI, the question of surgical exploration is always an underlying issue. In 1998, Pear et al. [2] concluded that pneumatosis in AMI “represents a surgical emergency.” However, a few years later, Kernagis et al. [3] and Wiesner et al. [4] already alerted that gas pattern was not a synonym of transmural necrosis in AMI. They presented a correlation between pathologic extension of necrosis in patients with a gas pattern and showed that the presence of gas “[did] not allow prediction of transmural bowel infarction.” Nevertheless, the idea that a gas pattern in AMI requires urgent surgical exploration remains. We propose several explanations. First, gas is associated in the collective imagination with necrosis, gangrene, and putrefaction. Gas pattern frightens physicians and surgeons. Second, until recently, no other feature was clearly associated with transmural necrosis (except for the pneumoperitoneum, of course). Recent evidence addresses this issue.

In AMI, a gas pattern is the consequence of mural necrosis that allows gas to pass from the mucosal to the submucosal space and eventually to bowel veins. Therefore, in occlusive AMI, pneumatosis indicates that bowel ischemia has begun, the occurrence of transmural necrosis being a matter of hours. One well-recognized downfall of this feature is its low sensitivity: pneumatosis occurs very late. In a prospective study of patients with occlusive AMI, our group identified bowel dilation as an independent predictor of necrosis requiring resection [5]. This has been validated internally since (submitted for publication). The bowel dilation is explained by ischemic lesions to the myenteric plexi, consistent with a late form of ischemia, and is depicted before the occurrence of pneumatosis. It is important to stress that said suspicion of necrosis should be reinforced by clinical (organ failure) and laboratory tests (elevated serum lactate) that should be interpreted altogether [5]. Therefore, an isolated pneumatosis in the absence of bowel dilatation or laboratory anomalies should not be considered a sign of necrosis. Conversely, bowel dilation in patients with elevated serum lactate indicates the presence of necrosis, even without pneumatosis. Of note, bowel dilation cannot be used in NOMI because most critically ill patients suffer from gastrointestinal failure, receive parenteral nutrition, and suffer from paralytic ileus for multiple reasons [6].

**Fig. 1:**
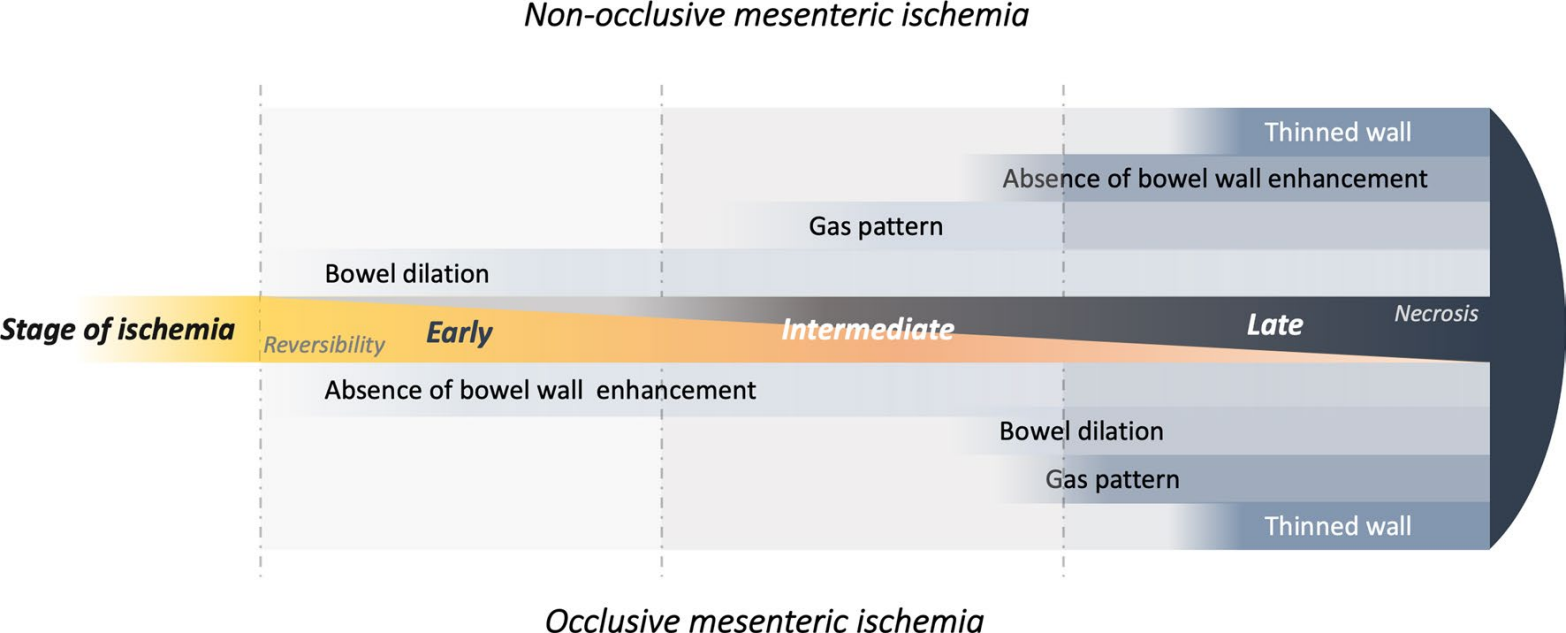
Time of onset of main CT features of acute mesenteric infarction according to the three main stages of bowel ischemia (i.e., early, intermediate, late). Differences between occlusive and non-occlusive mesenteric ischemia are presented

**Fig. 2:**
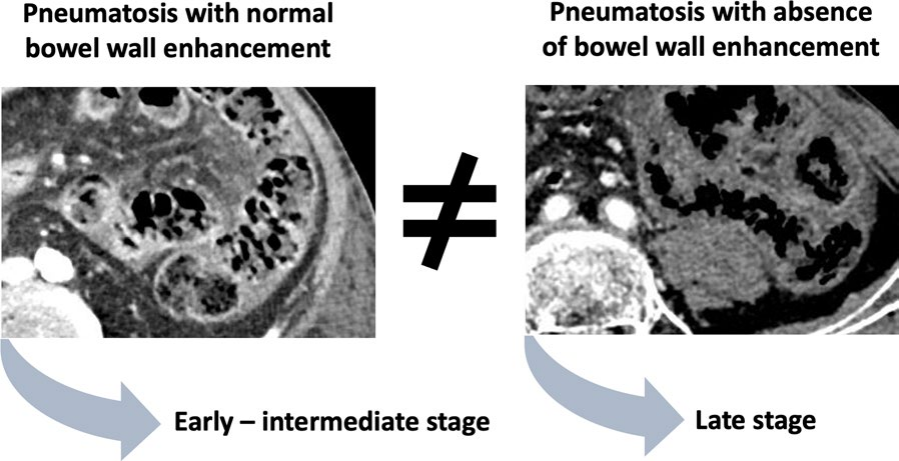
Representation of the two distinct situations in non-occlusive mesenteric ischemia when dealing with gas pattern. Analysis of bowel wall enhancement is mandatory even in the case of pneumatosis

In NOMI, pneumatosis should be considered a marker of current or recent bowel ischemia because it fails to assess the stage of AMI (Fig. 1). Our group recently showed that more consistent CT features should be searched for [6], namely a thinned wall (more specific) and an absence of bowel wall enhancement (more sensitive). Those signs should be assessed independently from the presence of pneumatosis to diagnose transmural necrosis (Fig. 2). In our recent study [7] of 154 patients with NOMI undergoing laparotomy, 81 underwent surgical exploration for suspected small bowel transmural necrosis. Among them, 31 (31/81, 38%) had gas pattern at CT. At the end, among these 31 patients with suspected small bowel NOMI associated with gas pattern, none of the patients (0/4) with normal wall enhancement had transmural necrosis. In contrast, 77% (21/27) of patients with gas patterns associated with an absence of bowel wall enhancement did. Here again, the meaning of pneumatosis shall be weighed against several other imaging and laboratory anomalies (Prothrombin time, serum bicarbonates).

## Conclusion

Pneumatosis will continue to be at the center of discussions in patients with AMI, as intensivists and surgeons will likely be bewitched by gas patterns. It is our role, as radiologists, to decipher this entity. If pneumatosis alone is considered, necrosis is likely to be overlooked in occlusive AMI and over diagnosed in NOMI. In all cases, more frequent, accurate, and valuable CT features of necrosis shall be searched for. Notably, the presence of a gas pattern should not be the sole reason for surgical exploration.

## Data Availability

All material is included in this Letter.

## Abbreviations

ABI: Acute bowel ischemia
AMI: Acute mesenteric infarction
CT: Computed tomography
NOMI: Non-occlusive mesenteric ischemia
OMI: Occlusive mesenteric ischemia

## References

[1] Davarpanah AH, Ghamari Khameneh A, Khosravi B, Mir A, Saffar H, Radmard AR (2021). Many faces of acute bowel ischemia: overview of radiologic staging. Insights Imaging.

[2] Pear BL (1998). Pneumatosis intestinalis: a review. Radiology.

[3] Kernagis LY, Levine MS, Jacobs JE (2003). Pneumatosis intestinalis in patients with ischemia: correlation of CT findings with viability of the bowel. AJR Am J Roentgenol.

[4] Wiesner W, Mortelé KJ, Glickman JN, Ji H, Ros PR (2001). Pneumatosis intestinalis and portomesenteric venous gas in intestinal ischemia: correlation of CT findings with severity of ischemia and clinical outcome. AJR Am J Roentgenol.

[5] On behalf of the SURVI group, Nuzzo A, Maggiori L et al (2017) Predictive factors of intestinal necrosis in acute mesenteric ischemia: prospective study from an intestinal stroke center. Am J Gastroenterol 112:597–605. 10.1038/ajg.2017.38 10.1038/ajg.2017.38 28266590

[6] Verdot P, Calame P, Winiszewski H (2021). Diagnostic performance of CT for the detection of transmural bowel necrosis in non-occlusive mesenteric ischemia. Eur Radiol.

[7] Calame P, Winiszewski H, Doussot A (2021). Evaluating the risk of irreversible intestinal necrosis among critically Ill patients with nonocclusive mesenteric ischemia. Am J Gastroenterol.

